# Hybrid Nanomaterials Based on Graphene and Gold Nanoclusters for Efficient Electrocatalytic Reduction of Oxygen

**DOI:** 10.1186/s11671-016-1552-0

**Published:** 2016-07-19

**Authors:** Changhong Wang, Na Li, Qiannan Wang, Zhenghua Tang

**Affiliations:** School of Materials and Energy, Guangdong University of Technology, Guangzhou, 510006 People’s Republic of China; New Energy Research Institute, School of Environment and Energy, South China University of Technology, Guangzhou Higher Education Mega Centre, Guangzhou, 510006 People’s Republic of China; Guangdong Provincial Key Laboratory of Atmospheric Environment and Pollution Control, Guangdong Provincial Engineering and Technology Research Center for Environmental Risk Prevention and Emergency Disposal, School of Environment and Energy, South China University of Technology, Guangzhou Higher Education Mega Centre, Guangzhou, 510006 People’s Republic of China

**Keywords:** Gold nanoclusters, Reduced graphene oxide, Nanocomposites, Electrocatalyst, Oxygen reduction reaction

## Abstract

Nanocomposites based on gold nanoclusters (AuNCs) with polyvinyl pyrrolidone as ligand and reduced graphene oxide (RGO) have been prepared and employed as efficient electrocatalysts for oxygen reduction reaction (ORR). AuNCs were synthesized through a wet chemical approach and then loaded onto the RGO. The as-prepared hybrid materials were pyrolyzed to remove the organic ligands. The composites were characterized by transmission electron microscopy (TEM), X-ray diffraction (XRD), and X-ray photoelectron spectroscopy (XPS) as well as other techniques. Electrochemical tests demonstrated that the hybrid materials exhibited effective ORR activity in alkaline media. Among a series of samples tested, the pyrolyzed sample with 50 % AuNCs mass loading exhibited the best activity, superior than AuNCs alone, RGO alone, and the others, in terms of onset potential and kinetic current density as well as durability. The method here may provide a generic approach to prepare supported noble metal nanoclusters with excellent reactivity and robust stability for ORR.

**Electronic supplementary material:**

The online version of this article (doi:10.1186/s11671-016-1552-0) contains supplementary material, which is available to authorized users.

## Background

Low-temperature fuel cells, an efficient energy conversion device, are expected to tackling the global energy crisis and environmental issues [1–3]. However, their massive commercialization and development have been significantly hindered by the oxygen reduction reaction (ORR), mainly due to the sluggish reaction kinetics and complicated reaction pathways of ORR [4, 5]. Currently, the most extensively employed catalyst for ORR is Pt-based materials [6–9], but the high price and scarcity of Pt as well as low poison resistance significantly retard their widespread commercialization. Therefore, it is urgent to develop cost-effective ORR catalyst with sufficient reactivity and high earth abundance as well as long-term stability [10–12].

Gold nanoclusters (AuNCs), owing to their excellent optical properties, and rich electrochemical behaviors as well as ultrasmall sizes imparted large surface area, have been attracting increasing research attentions in catalytic regime in the past decade [13]. Recent studies have demonstrated that AuNCs possess excellent catalytic activity toward a series of reaction including CO oxidation [14], selective hydrogenation [15], and oxidation [16] of small organic molecules as well as ORR [17]. Unfortunately, when employing AuNCs alone as catalyst, the stability became an inevitable issue, e.g., they can easily dissolve, aggregate, decompose, or sinter in the electrochemical catalytic process [18]. Consequently, a variety of substrates have been developed to support and stabilize these clusters, and the most widely used substrate is porous carbon materials including mesoporous carbon [19], carbon nanosheets [20], and carbon nanodots [21]. Graphene, although discovered only recently in 2004 [22], has already become an extremely promising material as a catalyst support [23]. Graphene-based materials (e.g., graphene, graphene oxide, reduced graphene oxide) possesses a two-dimensional sheet structure with sp^2^ hybridized carbon, which enables it with large surface area and excellent electric conductivity as well as great chemical stability in acidic or alkaline electrolytes [23, 24]. Notably, the sheet films made of reduced graphene have showed fast electron-transfer kinetics and remarkable electrocatalytic activity for a variety of electroactive species [25]. Molybdenum-doped mesoporous graphene composites have been prepared by Dong et al., and such composites exhibited excellent ORR activity through a four-electron dominant reaction pathway [26]. Surfactant-free Au clusters grown on reduced graphene oxide (RGO) sheets have been fabricated by the in situ approach, and such hybrid materials possessed a nearly close onset potential value with Pt/C in ORR [27]. Chen’s group reported a novel technique for the simultaneous synthesis of Au nanoparticle/RGO hybrid materials with enhanced ORR activity recently [28]. Despite the great success have been achieved, the most facile and straightforward method (mechanical mixing and calcination) have been ignored. Polyvinyl pyrrolidone (PVP) has been widely employed as a ligand or protecting agent to prepare monodisperse noble metal clusters with ultrasmall dimensions and narrow size distributions [29, 30]. The integration of PVP Au clusters and RGO by a simple and straightforward approach (mixing and calcination) might offer a promising alternative as ORR catalyst, and this is the primary motivation of our current study.

In this study, nanocomposites based on AuNCs with PVP as ligand and RGO have been prepared and employed as efficient electrocatalysts for ORR. Monodisperse AuNCs were prepared by a wet chemical approach and loaded onto RGO. The composites were calcined, and PVP ligands were removed. With low loading of AuNCs, the intimate interaction of RGO and AuNCs could prevent the agglomeration of AuNCs during pyrolysis, leading to the formation of uniform materials. At higher loading of AuNCs, obvious agglomeration was observed which resulted in diminished activity. All the hybrid materials demonstrated effective activity toward ORR. Among a series of samples tested, the sample with 50 % mass loading of AuNCs demonstrated best activity with a performance comparable with Pt/C, superior than AuNCs alone and RGO alone as well as other samples.

### Experimental Section

#### Materials

Hydrogen tetrachloroauric acid (III) trihydrate (HAuCl_4_ · 3H_2_O, 98 %, Energy Chemicals, Shanghai), polyvinyl pyrrolidone K30 (PVP K30, Boao Biotechnology Co. Ltd, Shanghai), sodium borohydride (NaBH_4_, 98 %, Aladdin industrial Corporation, Shanghai), Pt/C (20 wt%, Alfa Aesar), graphite powder (99.95 %, Aladdin industrial Corporation, Shanghai), concentrated sulfuric acid (concentrated H_2_SO_4_, 98 %, Dingshengxin, Tianjian), sodium nitrate (NaNO_3_, >99 %, Chemical Reagent Factory, Tianjin), potassium permanganate (KMnO_4_, >99.5, Aladdin industrial Corporation, Shanghai), hydrogen peroxide (H_2_O_2_, 30 %, Dingshengxin, Tianjian), hydrochloric acid (HCl, 36–38 %, Aladdin industrial Corporation, Shanghai), and hydrazine hydrate (H_6_N_2_O, >99.7 %, Dingshengxin, Tianjian) were used as received without further purification. Water was supplied by a Barnstead Nanopure Water System (18.3 MΩ cm).

### Preparation of PVP Stabilized Au Nanoclusters (PVP-AuNCs)

PVP-AuNCs were synthesized by following a modified procedure in the previous reports [29, 31]. Typically, 555.5 mg PVP was added to the tetrachloroauric acid (III) trihydrate (50 mM, 1 mL) solution and stirred for 30 min in an ice bath. Then, a freshly prepared sodium borohydride aqueous solution (0.1 M, 5 mL) was added under vigorously stirring. The solution color turned from light yellow into dark brown immediately, indicating the formation of PVP-AuNCs. The as-formed AuNCs were dialyzed by a semi-permeable membrane for 3 days to remove all the free ligands.

### Preparation of Reduced Graphene Oxide (RGO)

Graphene oxide (GO) was first acquired according to Hummer’s method [32]. Then, RGO was synthesized by following a reported protocol [27]. Briefly, 50 mg GO was dispersed in 50 mL water and sonicated for 1 h to obtain a brown suspension. Then, the dispersion was transferred into a three-neck flask, heated to 80 °C, and 1 mL hydrazine hydrate was added. The solution was kept stirring for 24 h and then filtered. The obtained solid was washed repeatedly with copious methanol and water and fully dried in a vacuum drying oven.

### Preparation of RGO Supported PVP-AuNCs

Twenty milligrams of RGO was dispersed in 20-mL water in a round bottom flask. Separately, 20 mg of AuNCs were added into 20-mL water under constant stirring for 20 min. Then, they were mixed with AuNCs-to-RGO mass ratios of 1:2, 1:1, and 2:1, respectively. The mixtures were sonicated for 3 h at room temperature. Subsequently, the solvents were removed by freeze drying, and the residual solids were calcined for 2 h under nitrogen stream at 600 °C to obtain the nanocomposite catalysts of RGO supported AuNCs.

### Characterizations

The ultraviolet visible absorption spectrum of AuNCs was tested through a Shimadzu 2600/2700 UV-visible scanning spectrophotometer. For high-resolution transmission electron microscopic (HR-TEM) tests, the samples were dispersed in absolute ethanol and dropcast directly onto a copper grid coated with a holey carbon film. The X-ray diffraction (XRD) were obtained with the Bragg angle (2 θ) changes in the scope of 10°–90° at room temperature by using Bruker D8 diffraction and Cu K alpha radiation (*λ* = 0.1541 nm). X-ray photoelectron spectroscopy (XPS) analysis was conducted with a VG MultiLab 2000 instrument with a monochromatic Al K X-ray source (Thermo VG Scientific).

### Electrochemical Measurements

The electrochemical tests were performed on an electrochemical workstation with the type of CHI 750E (CH instruments) in 0.1 M KOH solution at room temperature. The platinum wire electrode and Ag/AgCl electrode worked as the counter electrode and the reference electrode, respectively. The working electrode was a glassy carbon (5.61 mm in diameter) rotating ring disk electrode (37 % collection efficiency) from the pine instruments; the working electrode was cleaned with 0.3-um alumina powder on a polishing mica cloth.

Typically, 1-mg catalyst was dispersed in 0.5 mL of anhydrous ethanol solution, and 5-μL Nafion was added into the mixture and sonicated for at least half an hour. Ten-microliter mixed liquor was applied onto the glassy carbon electrode and dried at room temperature. The loading capacity of all catalyst samples on the electrode surface was 40.4 μg cm^–2^. In all tests, the Ag/AgCl reference electrode was calibrated with respect to a reversible hydrogen electrode (RHE). The calibration of the working electrode and the counter electrode was carried out in a highly pure H_2_ (99.999 %) saturated electrolyte using a platinum wire. The cyclic voltammograms (CV) were conducted at a scan rate of 10 mV s^–1^, and the linear sweep voltammograms (LSV) were operated with the rotation rates ranging from 100 to 2500 rpm. All electrochemical tests were conducted in 0.1 M KOH solution, *E*_RHE_ = *E*_Ag/AgCl_ + 0.966 V.

## Results and Discussion

**Fig. 1:**
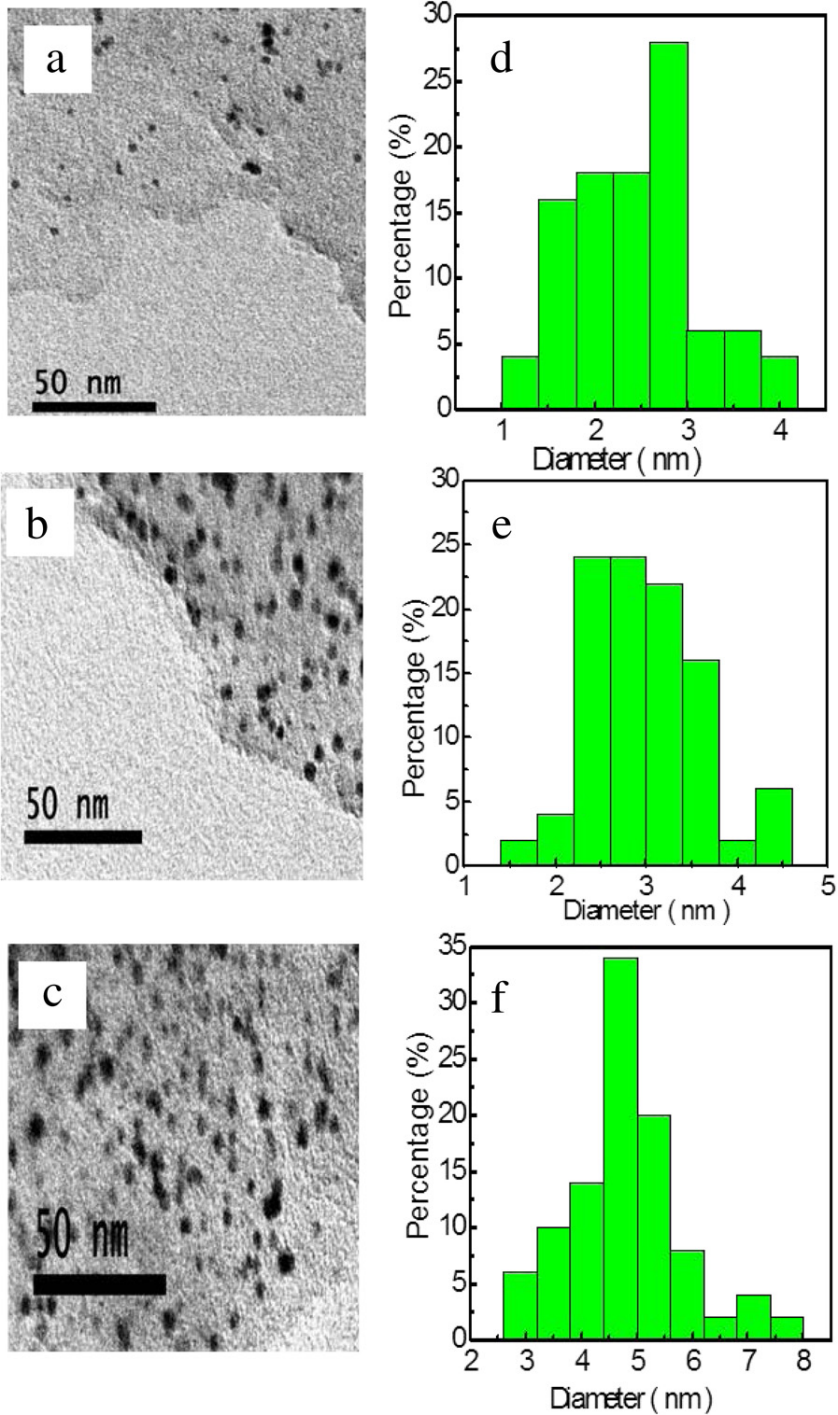
Representative TEM images and corresponding size distribution histogram of (**a**), **d** AuNCs:RGO = 1:2, **b**, **e** AuNCs:RGO = 1:1, and **c**, **f** AuNCs:RGO = 2:1

The AuNCs and RGO were first prepared separately. Additional file 1: Figure S1 presents the UV-visible absorbance spectrum of the as-prepared AuNCs. It is worth noting that, due to the well-known quantum confinement effects, gold nanoparticles with core diameter larger than 2.2 nm typically possess a surface plasmon resonance peak at ~520 nm in absorbance [13, 33]. Herein, the absorbance displays a featureless exponential decay without such peak, indicating that small gold clusters instead of large nanoparticles were acquired. Indeed, as shown in Additional file 1: Figure S2, the TEM results showed that the sample was very monodisperse, with an average diameter of 2.1 ± 0.2 nm. Representative TEM images of RGO can be found in Additional file 1: Figure S3, and the well-defined thin sheets can be easily identified. Then, the PVP-AuNCs were loaded into RGO, followed by pyrolysis at 600 °C for 2 h. Figure 1 presents the TEM results of the nanocomposites with the loading mass ratios for AuNCs-to-RGO of 1:2, 1:1, and 2:1, respectively. It can be observed that AuNCs were evenly distributed on the surface of RGO. Based on more than 200 counts of individual particles, the average diameters were calculated as 2.8 ± 0.3 nm, 2.4 ± 0.7 nm, and 4.7 ± 0.4 nm for 1:2, 1:1, and 2:1 samples, respectively. The core diameter was somewhat larger than that of the as-prepared PVP-AuNCs. Note that, the diameter of the sample of AuNCs:RGO = 2:1 is apparently larger than the other two, probably caused by the agglomeration during the pyrolysis resulted from the much higher AuNCs loading. Other TEM images for the nanocomposites of AuNCs:RGO = 1:1 with different magnitudes can be found in Additional file 1: Figure S4, which further confirm that AuNCs were evenly distributed on RGO.

**Fig. 2:**
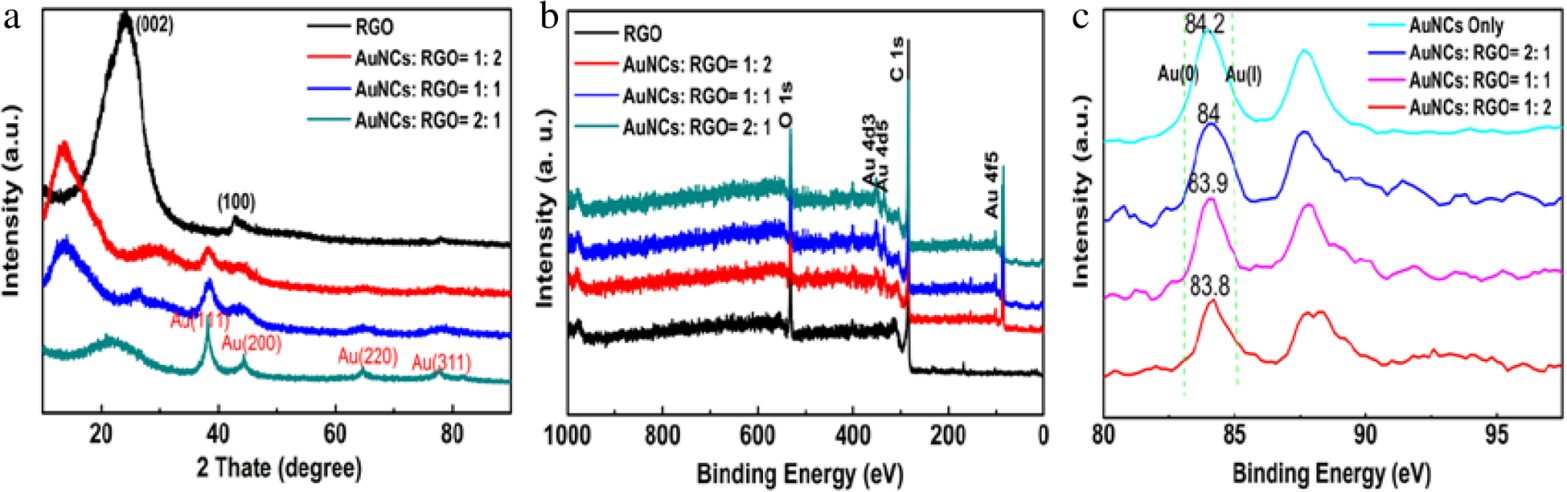
**a** XRD patterns, **b** XPS survey spectra, and **c** XPS Au 4f electron spectra of RGO and nanocomposite of AuNCs:RGO = 1:2, 1:1, and 2:1, respectively

The structure of the Au/RGO nanocomposites was further studied by XRD measurements. As shown in Fig. 2a, in the RGO sample, two peaks can be seen at 2θ = 23.9 and 43.3, which were assigned to (002) and (101) hexagonal carbon crystal face (jcpds75-1621), respectively. For AuNCs:RGO nanocomposites, additional diffraction peaks at 2θ = 38.5, 43.8, 64.7, and 78.8 can be observed, and these peaks correspond well with (111), (200), (220), and (311) diffraction of *fcc* gold [34]. The results implied that the PVP-AuNCs were indeed incorporated into the nanocomposite, which can be further attested by the XPS results in Fig. 2b. Besides the C1s peak and O1s peak from RGO, additional peaks from Au (Au4d, 353.19 and 333.12 eV, Au4f, 84.01 eV) with strong signal can be clearly identified. High-resolution XPS spectra of Au4f electrons of AuNCs and the nanocomposites can be found in Fig. 2c. For PVP-AuNCs, the binding energy of Au 4f_7/2_ is estimated as 84.2 eV, which is in the range of Au(0) film (83.8 eV) and Au(I) (85.0–86.0 eV) [35, 36]. While for the samples of nanocomposites, with the increasing of AuNCs, the binding energy decreased gradually from 84.0 eV for the 1:2 sample to 83.8 eV for the 2:1 samples. The binding energy decrease of Au 4f_7/2_ electrons indicated the electronic interaction between the gold elements and RGO, in good accordance with what we observed in the previous report [20]. Such electronic interaction might be beneficial for the fast electron transfer and mass transport kinetics during the electrochemical processes.

**Fig. 3:**
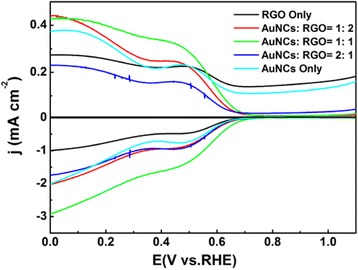
RRDE voltammograms of AuNCs, RGO, and composite catalysts with different AuNC loading in O_2_-saturated 0.1 M KOH solution at 2500 rpm

The electrocatalytic activity upon oxygen reduction of the nanocomposites was first examined by rotating ring disk electrode (RRDE) measurements. Figure 3 shows the RRDE results with the electrode modified by the nanocomposites of different AuNCs loadings (AuNCs:RGO = 1:2, 1:1, and 2:1) in an oxygen-saturated 0.1 M KOH solution at 2500 rpm. It can be observed that for all the samples, when the electrode potential was scanned to ~0.5 V, non-zero cathodic currents started to appear and reached a plateau at ~0.7 V. Such behavior suggests that these samples possess effective ORR activity. In addition, the voltammetric current of the ring electrode was about one order of magnitude lower than that of the disk electrode, which indicates that a relatively small amount of peroxide product was produced during the ORR. Note that, for AuNCs and RGO alone, the onset potentials are lower than the composites, while the voltammetric currents of the ring electrode are much higher than the composites, which suggests much more byproduct H_2_O_2_ was produced. Both of them highlight the merits of using hybrid materials. Interestingly, the catalyst performance varies with the change of AuNCs-to-RGO mass loading ratio. All the hybrid samples outperform than AuNCs and RGO alone in ORR, and apparently, the nanocomposite with AuNCs:RGO = 1:1 possessed superior reactivity than the other two samples. Similar results can also be found in CV measurement, which are shown in Additional file 1: Figure S5. The onset potential and diffusion-limited currents (at +0.55 V and 2500 rpm) can be estimated to 0.64 V and 0.97 mV cm^−2^ for AuNCs:RGO = 1:2, 0.8 V and 1.75 mV cm^−2^ for AuNCs:RGO = 1:1, and 0.67 V and 0.98 mV cm^−2^ for AuNCs:RGO = 2:1, respectively. The huge variation between the catalytic performance and different AuNCs loading suggest a delicate balance between the gold content and the effective surface area. In principle, the number of electrocatalytic active sites increased with the increasing of the AuNC loading, and this is why the performance for nanocomposite of AuNCs:RGO = 1:1 is better than that of AuNCs:RGO = 1:2. However, if the AuNCs are overloaded, agglomeration occurred during the pyrolysis, as observed in TEM measurements. The agglomeration may block some active sites, hence, significantly lower the ORR activity.

**Fig. 4:**
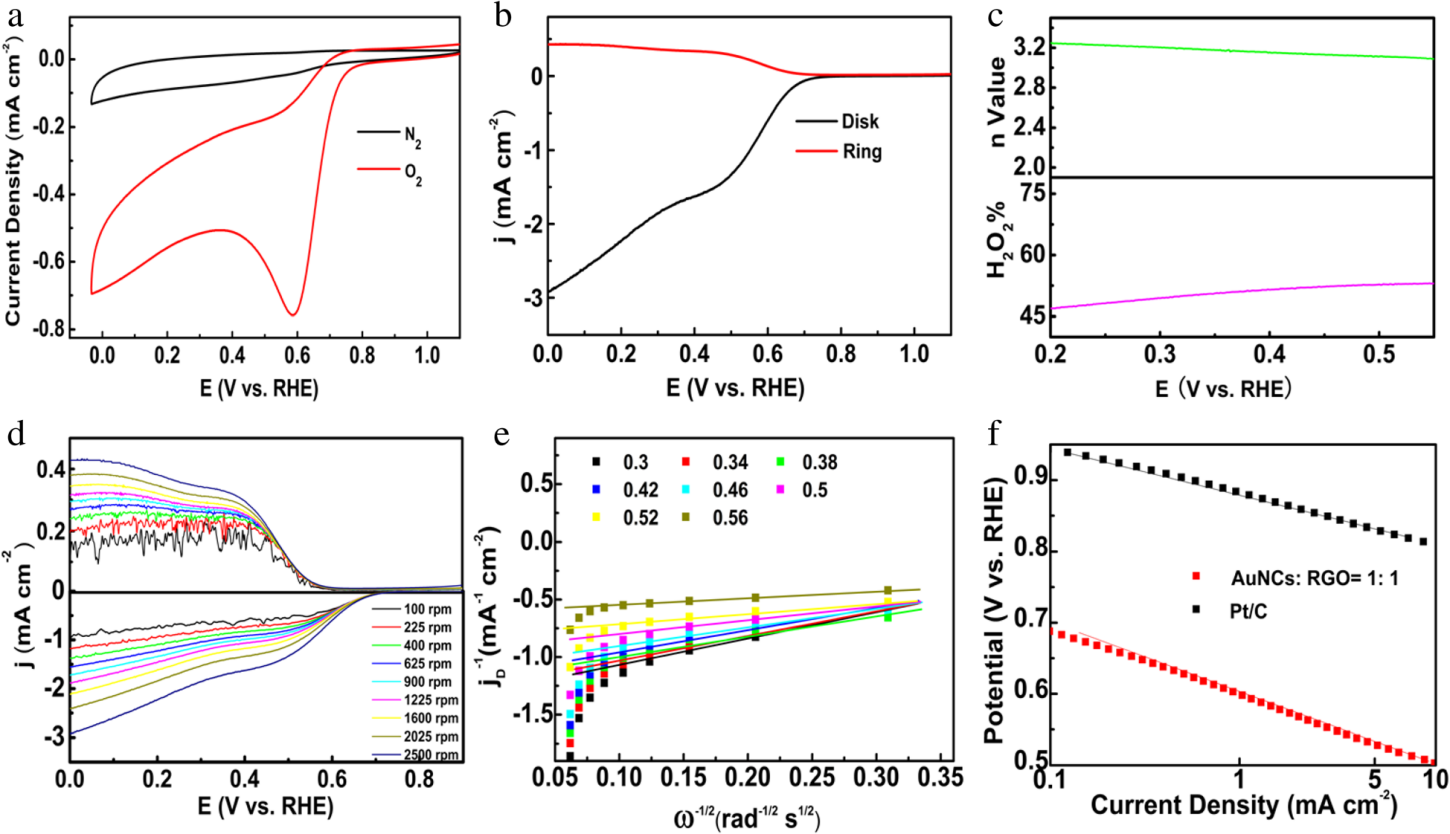
**a** Cyclic voltammograms, **b** RRDE voltammograms, **c** plots of H_2_O_2_ yield, and number of electron transfer of a glassy carbon electrode modified with nanocomposite of AuNCs:RGO (1:1) at the rotation speed of 2500 rpm. Statistic results were based on data of three independent measurements. **d** LSV curves for nanocomposite of AuNCs:RGO (1:1) at the rotation rates of 100 to 2500 rpm. **e** The corresponding K–L plots for nanocomposite of AuNCs:RGO (1:1) at different potentials. **f** The corresponding tafel plots for nanocomposite of AuNCs:RGO (1:1) and commercial Pt/C catalyst. All measurements were conducted at a catalyst loading of 40.4 μg/cm^2^ in an O_2_-saturated 0.1 M KOH aqueous solution with a sweep rate of 10 mV/s

Then, the electrocatalytic activity of the best sample (AuNCs:RGO = 1:1) was further tested by cyclic voltammetric method. As shown in Fig. 4a, in nitrogen-saturated 0.1 M KOH solution, only featureless double layer charging currents can be seen in the potential range from −0.04 to +1.16 V; however, in oxygen-saturated 0.1 M KOH solution, a sharp cathodic peak from oxygen reduction can be easily identified, indicating the effectiveness of the electrocatalytic activity of the sample for ORR. The RRDE voltammetric measurements can be found in Fig. 4b. The onset potential for the nanocomposites of AuNCs:RGO (1:1) is 0.80 V, while the diffusion-limited current is 1.75 mA cm^−2^ at 0.45 V and 2500 rpm.

According to the results of RRDE tests, the electron transfer number (*n*) and the yield of H_2_O_2_ in oxygen reduction process can be calculated by Equations (1) and (2):

1 $$ n=\frac{4{/}_d}{/_d+{/}_r/N} $$

2 $$ {\mathrm{H}}_2{\mathrm{O}}_2=\frac{200{I}_{\mathrm{r}}/N}{\frac{I_r}{N}+{I}_d} $$

in which, *I*_d_ is the disk current, *I*_r_ is the ring current, and *N* is the collection efficiency of RRDE (0.37). As can be seen from Fig. 3c, for nanocomposite of AuNCs:RGO (1:1), in the potential range from 0.2 to 0.55 V, the *n* value varied from 3.0 to 3.3, while the H_2_O_2_ yield changed from 46 to 49 %. The relatively low electron transfer number and high H_2_O_2_ yield indicated that the oxygen molecule probably took a partially 4e reduction pathway while some oxygen molecules were reduced to H_2_O_2_ but not directly to H_2_O [12, 17, 37].

Figure 4d presents the RRDE results of oxygen reduction for nanocomposites of AuNCs:RGO (1:1) collected with different rotation rates (from 100 to 2500 rpm) in O_2_-saturated 0.1 M KOH solution. One can see that the voltammetric current increased with the increasing of the rotation rates of the electrode. The corresponding Koutecky Levich (K-L) curve in Fig. 4e displayed a good linearity within the potential range of 0.3 to 0.56 V, implying a first-order reaction kinetics of ORR with respect to the oxygen concentration in the solution. Figure 4f shows the corresponding tafel curves for nanocomposite of AuNCs:RGO (1:1) (red curve) (60 mV dec^−1^) and Pt/C (black curve) (58 mV dec^−1^). Note that the two slopes are quite close, which implies that they exhibit similar reaction mechanism on the catalyst surface, where the rate determining step is probably the first electron transfer to oxygen molecule for both catalysts.

One can notice that the ORR activity of the nanocomposite catalysts was remarkably better than AuNCs or RGO alone, and the sample of AuNCs:RGO = 1:1 outperforms the other two composites. These results can be attributed into the following factors: First, the monodisperse PVP-AuNCs are very small with narrow size distribution, which favors for the activation for molecular oxygen [37–39]. As the AuNC loading increases, more effective catalytic sites are provided; however, overloading can cause agglomeration during pyrolysis, hence, lower the catalytic activity. Secondly, the RGO is probably more than a support but also plays an important role in the electronic interaction with AuNCs, evidenced by Au4f binding energy shift in the XPS results shown in Fig. 2b [27]. With appropriate AuNC loading, such interaction might prevent the migration and/or fusion of AuNCs in the RGO, hence, markedly enhanced the stability of the catalyst.

**Fig. 5:**
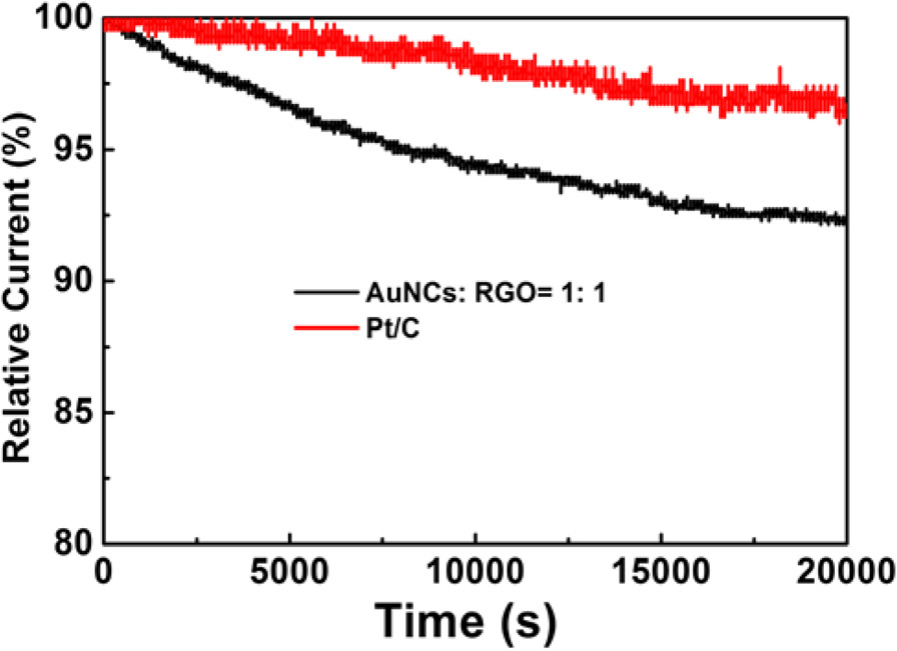
Chronoamperometric responses for ORR at AuNCs:RGO = 1:1 and Pt/C electrode in an O_2_-saturated 0.1 M KOH solution at +0.5 V for 20,000 s

Finally, the durability of the nanocomposites of AuNCs:RGO = 1:1 was examined by chronoamperometric measurements and compared with commercial Pt/C. As shown in Fig. 5, after more than 5 h of continuous operation, the cathodic current of the nanocomposite electrode only dropped to 94 %, with only a 6 % loss. Even if the currents of Pt/C only dropped 4 %, the durability of AuNCs:RGO (1:1) was still remarkable and close to Pt/C. Note that all the measurements were conducted with the hybrid catalysts which were recycled and tested three times.

## Conclusions

In this work, the composite catalysts of RGO supported AuNCs were fabricated and employed as efficient catalysts for ORR. The hybrid materials exhibited effective ORR catalytic activity in alkaline media. Among a series of samples, the composite with AuNCs:RGO = 1:1 demonstrated the best reactivity, within the context of onset potential and kinetic current density as well as durability. This work highlights the facile and straightforward approach to fabricate hybrid materials based on ultrasmall noble metal clusters with improved electrocatalytic performance. Further investigation with rational design of supported metal nanoclusters to achieve better electrocatalytic properties is still underway.

## Additional file

Additional file 1: Figure S1. UV-visible absorbance spectrum of PVP-AuNCs. **Figure S2.** Representative TEM images of PVP-AuNCs and their size distribution histogram. **Figure S3.** Representative TEM images of RGO. **Figure S4.** Representative TEM images with different magnitudes for nanocomposite of RGO/AuNCs (1:1). **Figure S5.** CV measurements of nanocomposites with different AuNCs loadings in O_2_-saturated 0.1 M KOH at a scanning rate of 10 mV/s. (DOC 16377 kb)

## References

[1] Debe MK (2012). Electrocatalyst approaches and challenges for automotive fuel cells. Nature.

[2] Kraytsberg A, Ein-Eli Y (2014). Review of advanced materials for proton exchange membrane fuel cells. Energy Fuels.

[3] Gasteiger HA, Marković NM (2009). Just a dream—or future reality?. Science.

[4] Swider-Lyons KE, Campbell SA (2013). Physical chemistry research toward proton exchange membrane fuel cell advancement. J Phys Chem Lett.

[5] Gewirth AA, Thorum MS (2010). Electroreduction of dioxygen for fuel-cell applications: materials and challenges. Inorg Chem.

[6] Guo S, Zhang S, Sun S (2013). Tuning nanoparticle catalysis for the oxygen reduction reaction. Angew Chem Int Ed.

[7] Bing Y, Liu H, Zhang L, Ghosh D, Zhang J (2010). Nanostructured Pt-alloy electrocatalysts for PEM fuel cell oxygen reduction reaction. Chem Soc Rev.

[8] Lim B, Jiang M, Camargo PHC, Cho EC, Tao J, Lu X, Zhu Y, Xia Y (2009). Pd-Pt bimetallic nanodendrites with high activity for oxygen reduction. Science.

[9] Stamenkovic VR, Fowler B, Mun BS, Wang G, Ross PN, Lucas CA, Marković NM (2007). Improved oxygen reduction activity on Pt3Ni(111) via increased surface site availability. Science.

[10] Zhang P, Sun F, Xiang Z, Shen Z, Yun J, Cao D (2014). ZIF-derived in situ nitrogen-doped porous carbons as efficient metal-free electrocatalysts for oxygen reduction reaction. Energy Environ Sci.

[11] Wu G, Zelenay P (2013). Nanostructured nonprecious metal catalysts for oxygen reduction reaction. Acc Chem Res.

[12] Niu W, Li L, Liu X, Wang N, Liu J, Zhou W, Tang Z, Chen S (2015). Mesoporous N-doped carbons prepared with thermally removable nanoparticle templates: an efficient electrocatalyst for oxygen reduction reaction. J Am Chem Soc.

[13] Murray RW (2008). Nanoelectrochemistry: metal nanoparticles, nanoelectrodes, and nanopores. Chem Rev.

[14] Herzing AA, Kiely CJ, Carley AF, Landon P, Hutchings GJ (2008). Identification of active gold nanoclusters on iron oxide supports for CO oxidation. Science.

[15] Zhu Y, Qian H, Drake BA, Jin R (2010). Atomically precise Au(25)(SR)(18) nanoparticles as catalysts for the selective hydrogenation of alpha,beta-unsaturated ketones and aldehydes. Angew Chem Int Ed.

[16] Turner M, Golovko VB, Vaughan OPH, Abdulkin P, Berenguer-Murcia A, Tikhov MS, Johnson BFG, Lambert RM (2008). Selective oxidation with dioxygen by gold nanoparticle catalysts derived from 55-atom clusters. Nature.

[17] Chen W, Chen S (2009). Oxygen electroreduction catalyzed by gold nanoclusters: strong core size effects. Angew Chem Int Ed.

[18] Tang W, Lin H, Kleiman-Shwarsctein A, Stucky GD, McFarland EW (2008). Size-dependent activity of gold nanoparticles for oxygen electroreduction in alkaline electrolyte. J Phys Chem C.

[19] Wang S, Zhao Q, Wei H, Wang J-Q, Cho M, Cho HS, Terasaki O, Wan Y (2013). Aggregation-free gold nanoparticles in ordered mesoporous carbons: toward highly active and stable heterogeneous catalysts. J Am Chem Soc.

[20] Wang Q, Wang L, Tang Z, Wang F, Yan W, Yang H, Zhou W, Li L, Kang X, Chen S (2016). Oxygen reduction catalyzed by gold nanoclusters supported on carbon nanosheets. Nanoscale.

[21] Liu M, Chen W (2013). Green synthesis of silver nanoclusters supported on carbon nanodots: enhanced photoluminescence and high catalytic activity for oxygen reduction reaction. Nanoscale.

[22] Novoselov KS, Geim AK, Morozov SV, Jiang D, Zhang Y, Dubonos SV, Grigorieva IV, Firsov AA (2004). Electric field effect in atomically thin carbon films. Science.

[23] Liu M, Zhang R, Chen W (2014). Graphene-supported nanoelectrocatalysts for fuel cells: synthesis, properties, and applications. Chem Rev.

[24] Chen D, Feng H, Li J (2012). Graphene oxide: preparation, functionalization, and electrochemical applications. Chem Rev.

[25] Tang L, Wang Y, Li Y, Feng H, Lu J, Li J (2009). Preparation, structure, and electrochemical properties of reduced graphene sheet films. Adv Funct Mater.

[26] Dong Y, Liu M, Liu Y, Wang S, Li J (2015). Molybdenum-doped mesoporous carbon/graphene composites as efficient electrocatalysts for the oxygen reduction reaction. J Mater Chem A.

[27] Yin H, Tang H, Wang D, Gao Y, Tang Z (2012). Facile synthesis of surfactant-free Au cluster/graphene hybrids for high-performance oxygen reduction reaction. ACS Nano.

[28] Govindhan M, Chen A (2015). Simultaneous synthesis of gold nanoparticle/graphene nanocomposite for enhanced oxygen reduction reaction. J Power Source.

[29] Tsunoyama H, Tsukuda T (2009). Magic numbers of gold clusters stabilized by PVP. J Am Chem Soc.

[30] Tsunoyama H, Tsukuda T, Sakurai H (2007). Synthetic application of PVP-stabilized an nanocluster catalyst to aerobic oxidation of alcohols in aqueous solution under ambient conditions. Chem Lett.

[31] Tsunoyama H, Sakurai H, Negishi Y, Tsukuda T (2005). Size-specific catalytic activity of polymer-stabilized gold nanoclusters for aerobic alcohol oxidation in water. J Am Chem Soc.

[32] Titelman GI, Gelman V, Bron S, Khalfin RL, Cohen Y, Bianco-Peled H (2005). Characteristics and microstructure of aqueous colloidal dispersions of graphite oxide. Carbon.

[33] Varnavski O, Ramakrishna G, Kim J, Lee D, Goodson T (2010). Critical size for the observation of quantum confinement in optically excited gold clusters. J Am Chem Soc.

[34] Qian H, Jin R (2009). Controlling nanoparticles with atomic precision: the case of Au(144)(SCH(2)CH(2)Ph)(60). Nano Lett.

[35] Tang Z, Xu B, Wu B, Germann MW, Wang G (2010). Synthesis and structural determination of multidentate 2,3-dithiol-stabilized Au clusters. J Am Chem Soc.

[36] Tang Z, Robinson DA, Bokossa N, Xu B, Wang S, Wang G (2011). Mixed dithiolate durene-DT and monothiolate phenylethanethiolate protected Au130 nanoparticles with discrete core and core-ligand energy states. J Am Chem Soc.

[37] Liu X, Li L, Zhou W, Zhou Y, Niu W, Chen S (2015). High-performance electrocatalysts for oxygen reduction based on nitrogen-doped porous carbon from hydrothermal treatment of glucose and dicyandiamide. ChemElectroChem.

[38] He G, Song Y, Liu K, Walter A, Chen S, Chen S (2013). Oxygen reduction catalyzed by platinum nanoparticles supported on graphene quantum dots. ACS Catal.

[39] Song Y, Chen S (2014). Graphene quantum-dot-supported platinum nanoparticles: defect-mediated electrocatalytic activity in oxygen reduction. ACS Appl Mater Interfaces.

