# Supplementary material for: Hybrid Nanomaterials Based on Graphene and Gold Nanoclusters for Efficient Electrocatalytic Reduction of Oxygen
Source: Nanoscale Res Lett. 2016 Jul 19;11:336. doi: 10.1186/s11671-016-1552-0 (PMC4949206; doi:10.1186/s11671-016-1552-0)
Supplement: Additional file 1: Figure S1. — UV-visible absorbance spectrum of PVP-AuNCs. Figure S2. Representative TEM images of PVP-AuNCs and their size distribution histogram. Figure S3. Representative TEM images of RGO. Figure S4. Representative TEM images with different magnitudes for nanocomposite of RGO/AuNCs (1:1). Figure S5. CV measurements of nanocomposites with different AuNCs loadings in O2-saturated 0.1 M KOH at a scanning rate of 10 mV/s. (DOC 16377 kb) [file 11671_2016_1552_MOESM1_ESM.doc]

Supplementary Materials

**Hybrid Nanomaterials Based on Graphene and Gold Nanoclusters for Efficient Electrocatalytic Reduction of Oxygen**

Changhong Wang,^1, *^ Na Li,^1, 2^ Qiannan Wang,^2^ Zhenghua Tang^*,2,3^

1 School of Materials and Energy, Guangdong University of Technology, Guangzhou, 510006, P. R. China. Email: wangchh@gdut.edu.cn

2 New Energy Research Institute, School of Environment and Energy, South China University of Technology, Guangzhou Higher Education Mega Centre, Guangzhou, 510006, P. R. China. Email: zhht@scut.edu.cn

3 Guangdong Provincial Key Laboratory of Atmospheric Environment and Pollution Control, Guangdong Provincial Engineering and Technology Research Center for Environmental Risk Prevention and Emergency Disposal, School of Environment and Energy, South China University of Technology, Guangzhou Higher Education Mega Centre, Guangzhou, 510006, P. R. China

**Figure S1**. UV-visible absorbance spectrum of PVP-AuNCs.


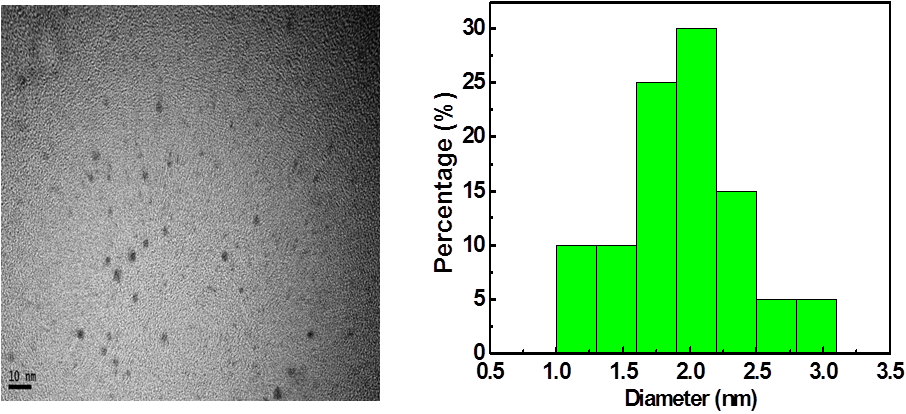


**Figure S2**. Representative TEM images of PVP-AuNCs and their size distribution histogram.


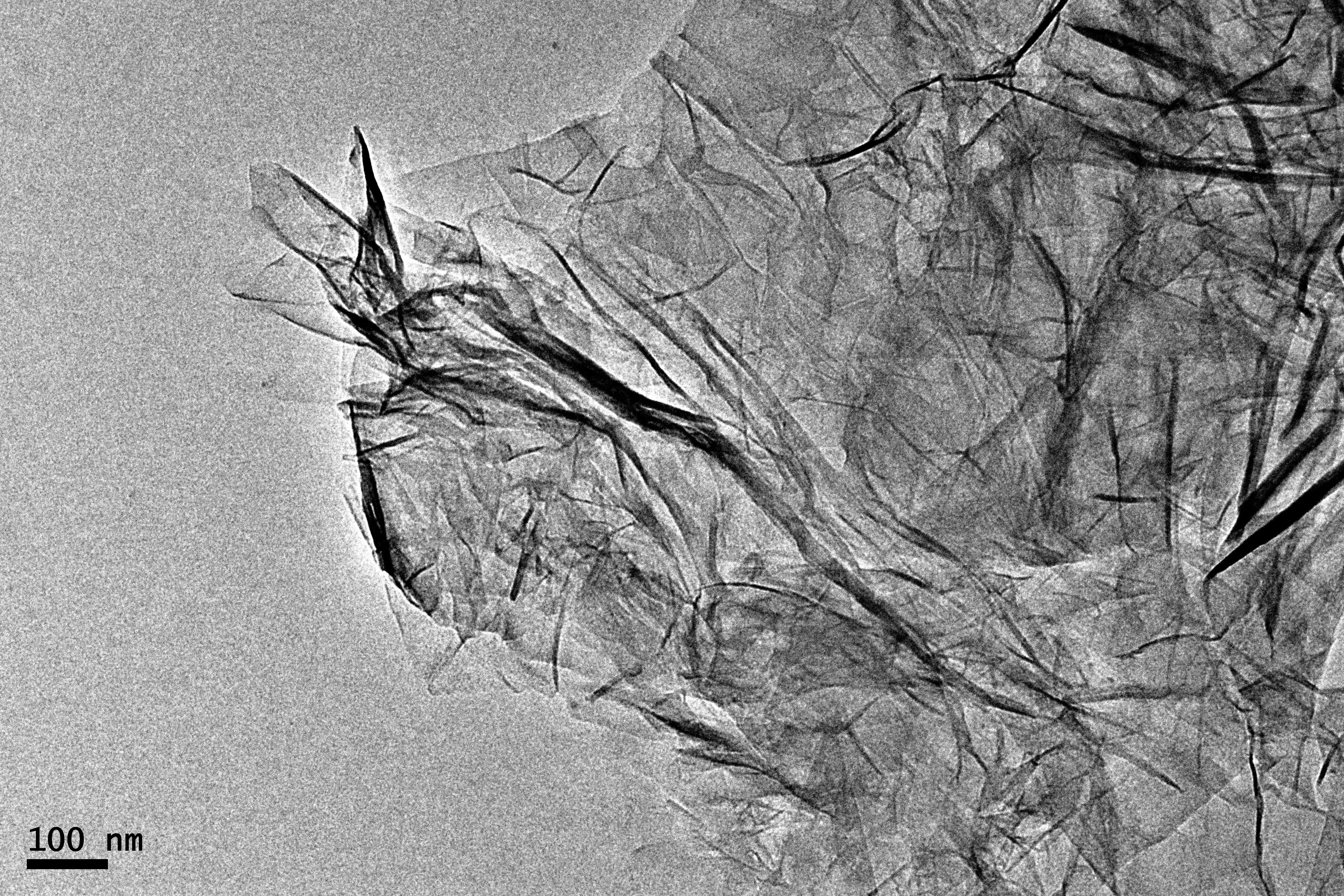


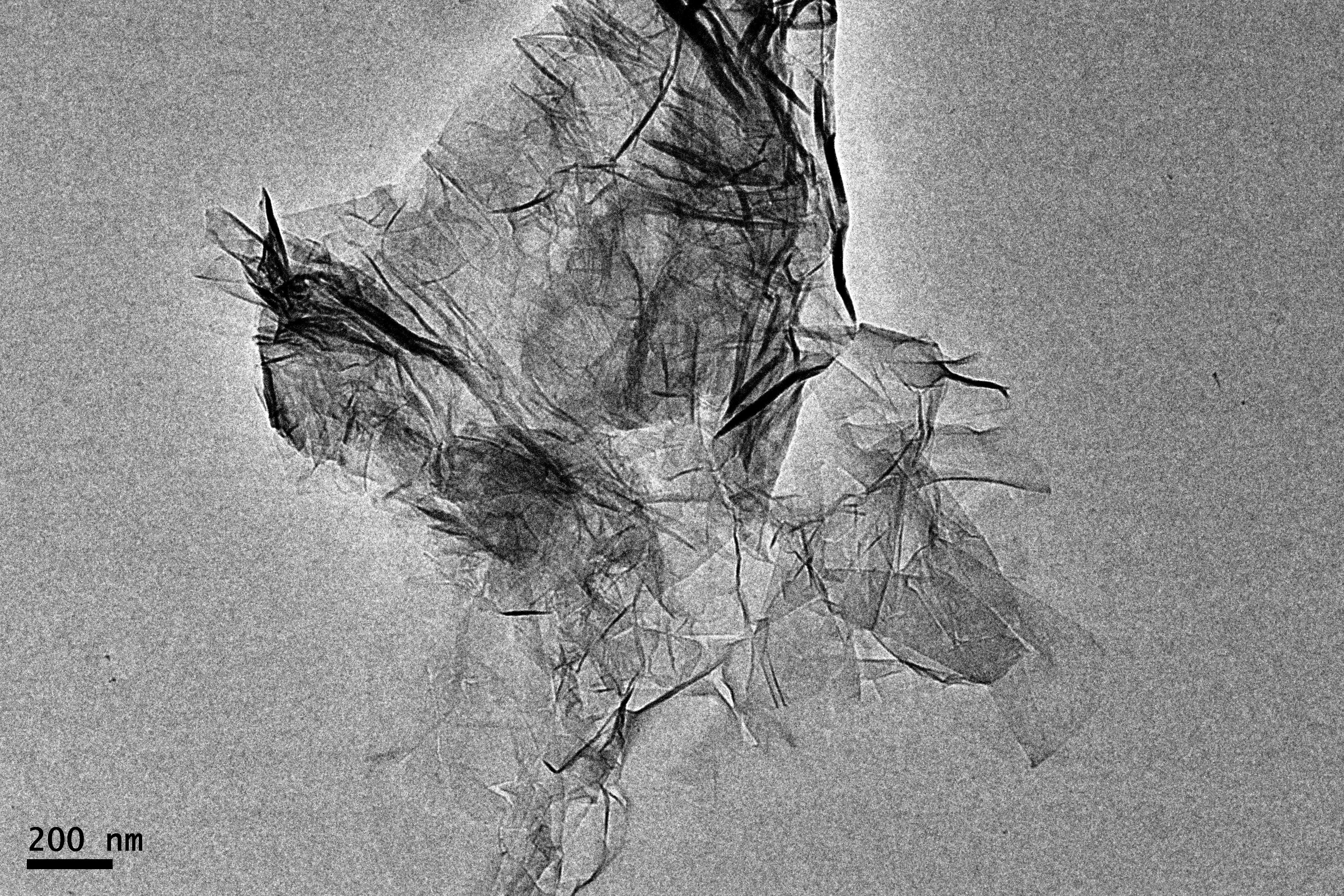


**Figure S3**. Representative TEM images of RGO.


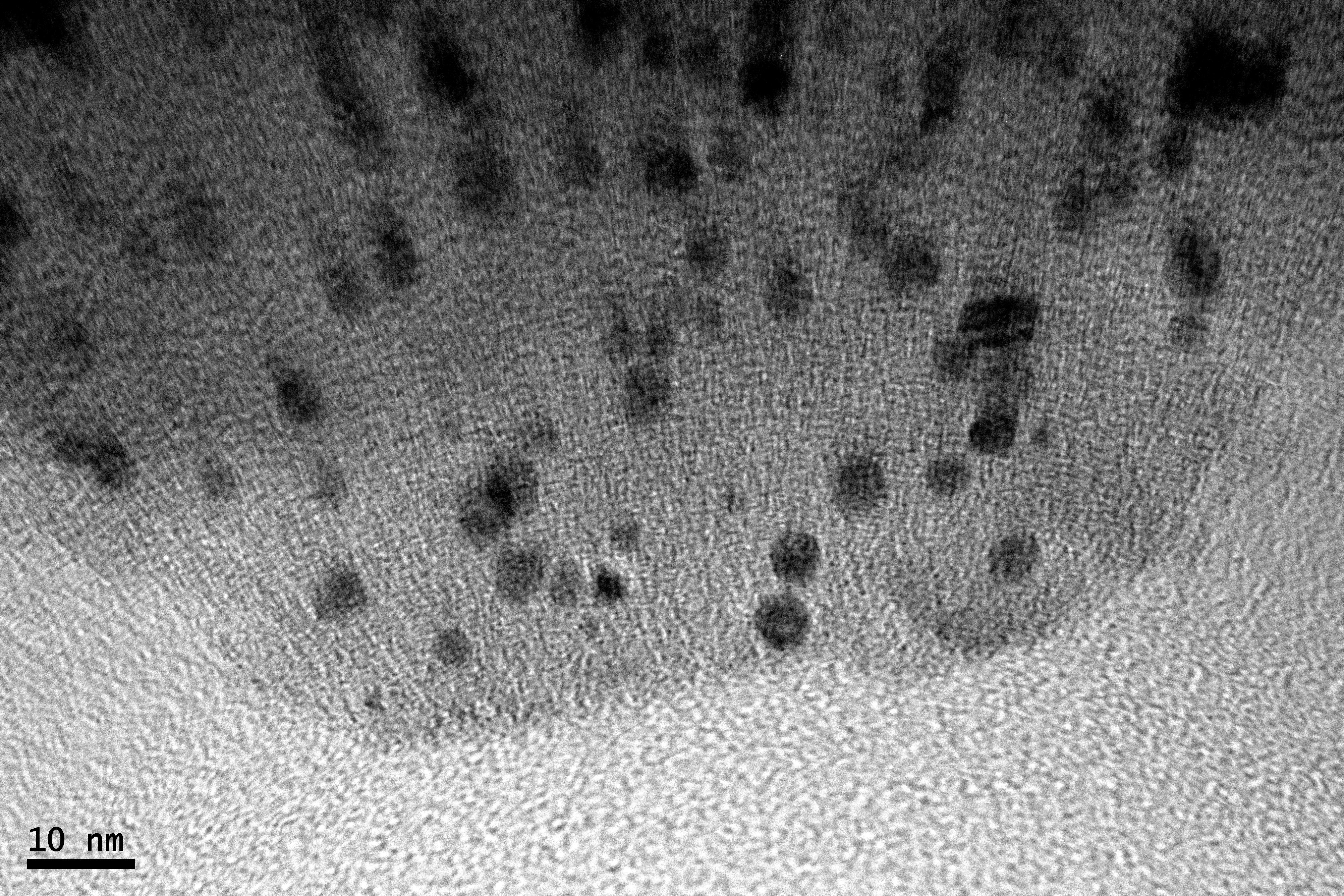

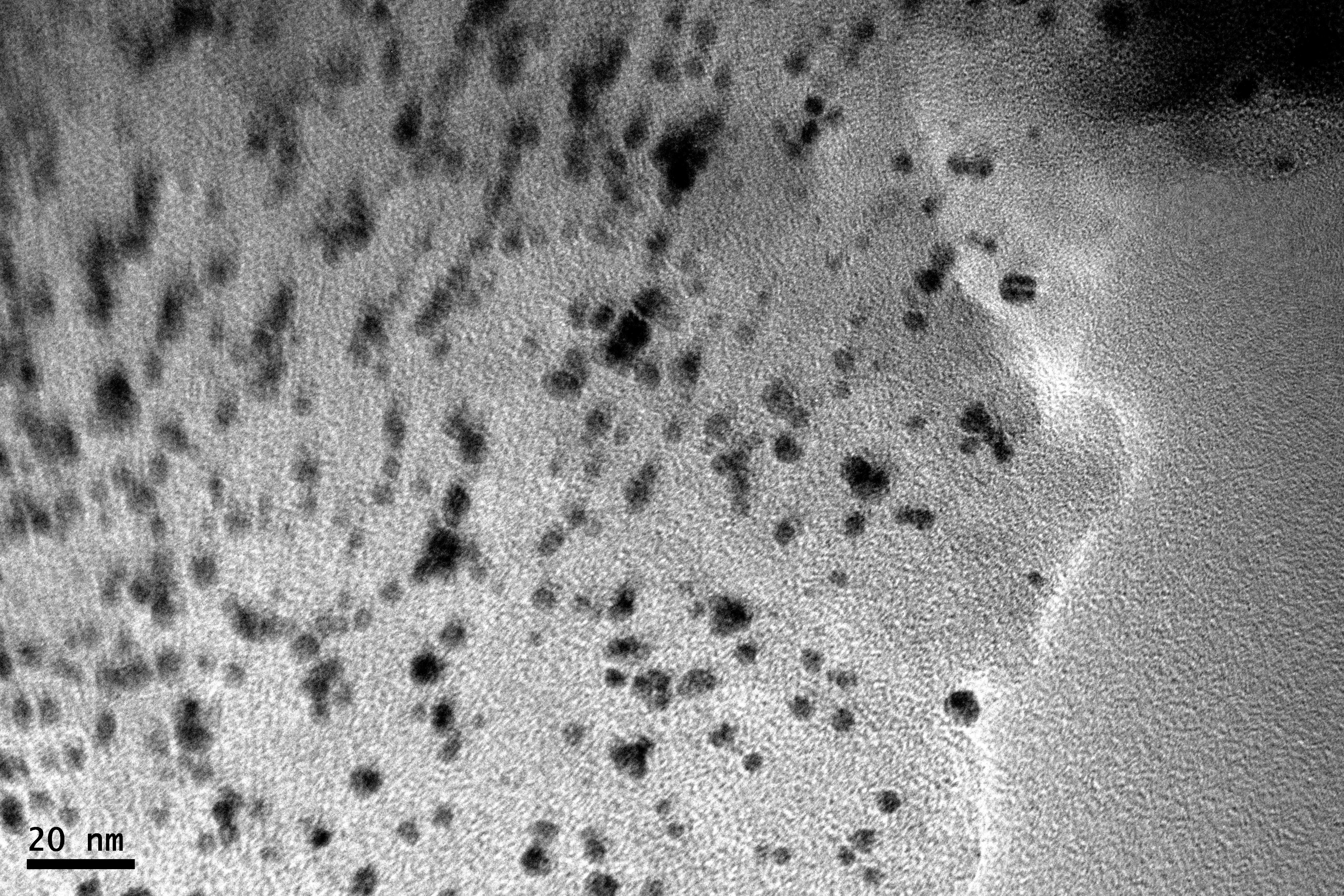


**Figure S4**. Representative TEM images with different magnitudes for nanocomposite of RGO/AuNCs (1: 1).

**Figure S5**. CV measurements of nanocomposites with different AuNCs loadings in O_2_-saturated 0.1 M KOH at a scanning rate of 10 mV/s.
